# Crystallite size-dependent metastable phase formation of TiAlN coatings

**DOI:** 10.1038/s41598-017-16567-z

**Published:** 2017-11-23

**Authors:** Marcus Hans, Denis Music, Yen-Ting Chen, Lena Patterer, Anders O. Eriksson, Denis Kurapov, Jürgen Ramm, Mirjam Arndt, Helmut Rudigier, Jochen M. Schneider

**Affiliations:** 1 Materials Chemistry, RWTH Aachen University, Kopernikusstraße 10, D-52074 Aachen, Germany; 2 Oerlikon Surface Solutions AG, Oerlikon Balzers, Iramali 18, LI-9496 Balzers, Liechtenstein; 3 Oerlikon Surface Solutions AG, Oerlikon Balzers, Churer Strasse 120, CH-8808 Pfäffikon, Switzerland; 4 Present Address: Center for Solvation Science, Ruhr-Universität Bochum, Universitätsstraße 150, D-44801 Bochum, Germany

## Abstract

It is well known that surface energy differences thermodynamically stabilize nanocrystalline γ-Al_2_O_3_ over α-Al_2_O_3_. Here, through correlative *ab initio* calculations and advanced material characterization at the nanometer scale, we demonstrate that the metastable phase formation of nanocrystalline TiAlN, an industrial benchmark coating material, is crystallite size-dependent. By relating calculated surface and volume energy contributions to the total energy, we predict the chemical composition-dependent phase boundary between the two metastable solid solution phases of cubic and wurzite Ti_1−x_Al_x_N. This phase boundary is characterized by the critical crystallite size *d*_*critical*_. Crystallite size-dependent phase stability predictions are in very good agreement with experimental phase formation data where *x* was varied by utilizing combinatorial vapor phase condensation. The wide range of critical Al solubilities for metastable cubic Ti_1−x_Al_x_N from *x*_max_ = 0.4 to 0.9 reported in literature and the sobering disagreement thereof with DFT predictions can at least in part be rationalized based on the here identified crystallite size-dependent metastable phase formation. Furthermore, it is evident that predictions of critical Al solubilities in metastable cubic TiAlN are flawed, if the previously overlooked surface energy contribution to the total energy is not considered.

## Introduction

**Figure 1:**
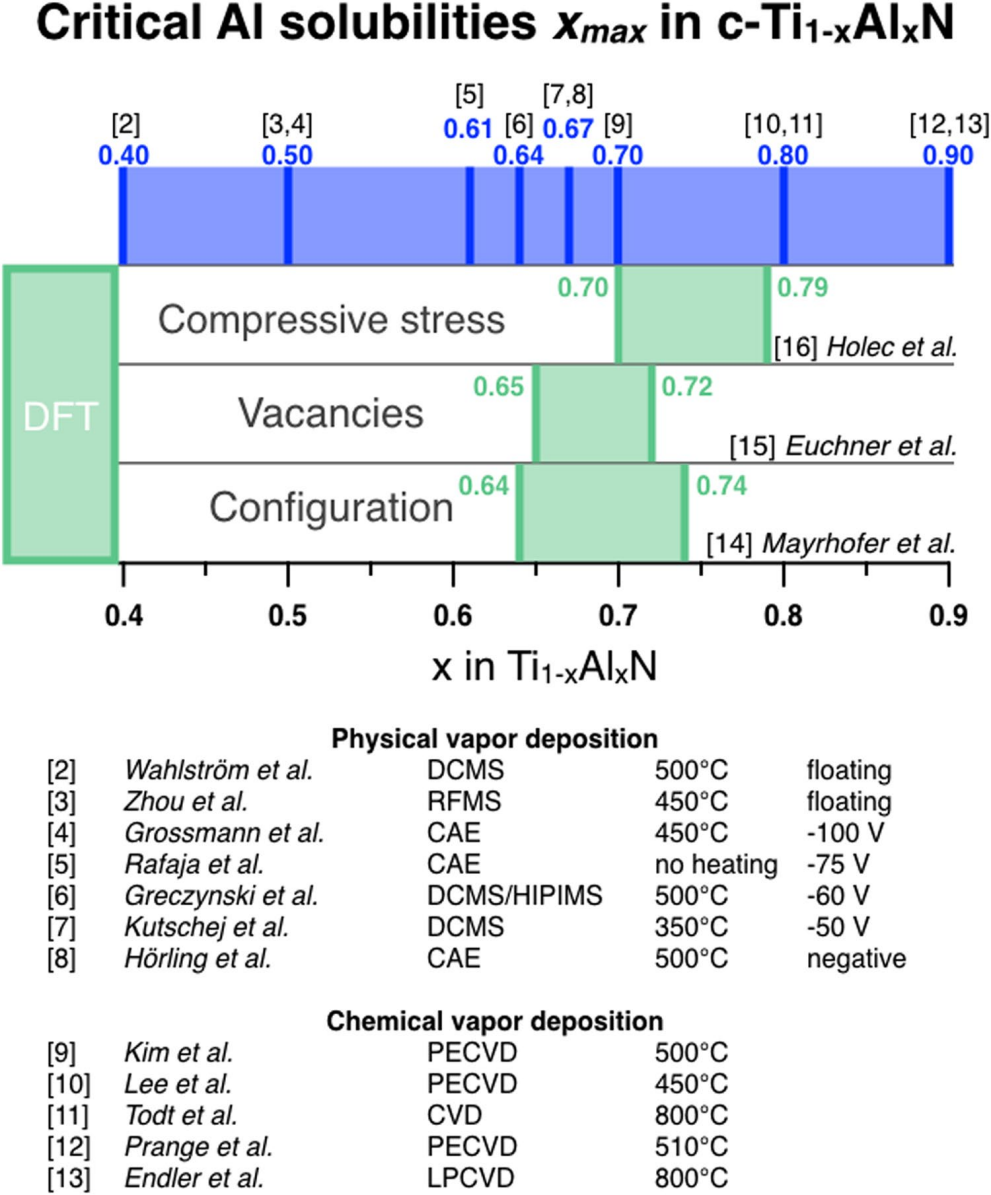
Comparison of *ab initio* calculations-based predicted critical Al solubilities *x*_*max*_ in metastable c-Ti_1−x_Al_x_N with growth experiments. The reference number corresponds to the number within the section References. First author names are provided together with the vapor condensation technique (DCMS = direct current magnetron sputtering, RFMS = radio frequency magnetron sputtering, CAE = cathodic arc evaporation, HIPIMS = high power impulse magnetron sputtering, PECVD = plasma-enhanced chemical vapor deposition, LPCVD = low pressure chemical vapor deposition), growth temperature as well as substrate bias potential.

While material property predictions based on density functional theory (DFT) calculations have been shown to be valuable in guiding materials design efforts^1^, an honest appraisal of the predictive capabilities regarding metastable phase formation is a sobering experience. Specifically for nanocrystalline metastable cubic (c-)Ti_1−x_Al_x_N, an industrial benchmark hard coating, critical Al solubilities of *x*_*max*_ = 0.4 to 0.9 were reported^2–13^ (see Fig. 1), while for larger Al concentrations the formation of metastable wurtzite (w-)TiAlN phase is observed. In contrast to the experimental data, the *x*_*max*_ range for metastable c-Ti_1−x_Al_x_N of only 0.64 to 0.79 is predicted by DFT calculations, considering explicitly compositional configurations^14^, vacancies on the metal and non-metal sublattice^15^ as well as compressive stresses^16^. The published DFT predictions are state of the art and within DFT accuracy correct. Although these predictions are relevant, useful and have provided guidance for experiments, the comparison in Fig. 1 demonstrates that 70% of the published experimental critical Al solubility data can not be predicted with these calculations.

McHale *et al*. have shown that differences in surface energy can stabilize γ-Al_2_O_3_ over α-Al_2_O_3_ by comparing molecular dynamics^17^ with calorimetric data^18^ for these alumina polymorphs at various crystallite sizes. Stability ranges were determined by identifying the crossover of the enthalpy as a function of surface area curves for thermodynamically stable γ- and α-Al_2_O_3_ phases^18^. The critical crystallite size *d*_*critical*_ depends directly on the crossover surface area *A*_*surf-crossover*_ and the density *ρ* (details on the physical origin of equation (1) are provided in the Methods section):

1 $${d}_{critical}=\frac{6}{\rho {A}_{surf \mbox{-} crossover}}$$

Crystallite size-dependent phase boundaries were also reported for thermodynamically stable ZrO_2_^19^ and TiO_2_^20^ phases. In contrast to thermodynamically stable phases, nanocrystalline metastable c-TiAlN is formed by kinetically limited growth from the vapor phase at cooling rates of up to 10^15^ K s^−1^ (ref.^21^). From the discussion above it is evident that surface energy differences can favor the formation of a particular phase^18^. Furthermore, the fact that surface energies can affect the critical solubility is well known from text book materials thermodynamics as Gibbs-Thomson effect^22^. Therefore, it is reasonable to assume that the crystallite size also influences the phase formation of metastable materials. This hypothesis was appraised critically by correlative *ab initio* calculations and spatially-resolved compositional as well as structural analysis of combinatorially grown metastable Ti_1−x_Al_x_N coatings.

## Results and Discussion

**Figure 2:**
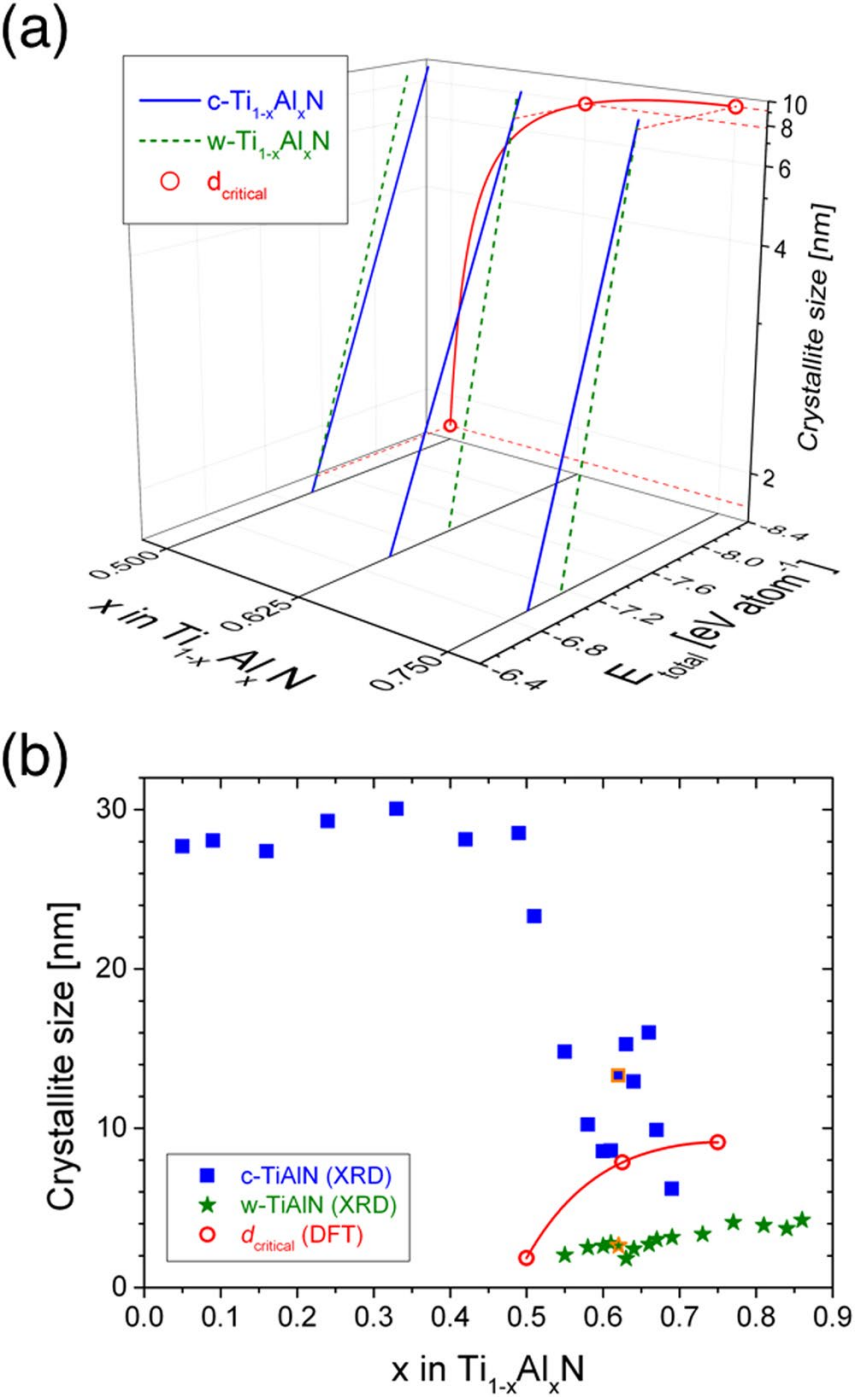
Calculated critical crystallite sizes and comparison with experimental data for Ti_1−x_Al_x_N. (**a**) Predicted *d*_*critical*_ values of Ti_0.5_Al_0.5_N, Ti_0.375_Al_0.625_N and Ti_0.25_Al_0.75_N. Solid and dashed lines correspond to total energy values of c- and w-Ti_1−x_Al_x_N, respectively. (**b**) Comparison of *d*_*critical*_ to crystallite sizes of Ti_1−x_Al_x_N coatings. Filled squares, filled stars and open circles represent experimental crystallite sizes of c- and w-Ti_1−x_Al_x_N and critical crystallite sizes, obtained by DFT. The solid line connecting *d*_*critical*_ data serves as a guide to the eye. Data points with an orange frame indicate the selected Ti_0.38_Al_0.62_N coating for spatially-resolved characterization provided in Fig. 3.

The chemical composition-dependent values of the critical crystallite size *d*_*critical*_ were calculated by DFT at the crossover of c- and w-Ti_1−x_Al_x_N total energies and are presented in Fig. 2(a). Hence, the chemical composition-dependent phase boundary between the two metastable solid solution phases of c- and w-TiAlN is defined by *d*_*critical*_. The stability range for c-TiAlN is defined by the crystallite size of c-TiAlN (*d*_*c*_) ≥ *d*_*critical*_ (*E*_*total,cubic*_ ≤ *E*_*total,wurtzite*_) while the stability range for w-TiAlN is determined by the crystallite size of w-TiAlN (*d*_*w*_) ≤ *d*_*critical*_ (*E*_*total,cubic*_ ≥ *E*_*total,wurtzite*_). At an Al concentration of 25 at.% (*x* = 0.50) the cubic phase is predicted to be stable for crystallite sizes *d*_*c*_ ≥ *d*_*critical*_ = 1.9 nm, while for the larger Al concentrations of 31.25 at.% (*x* = 0.625) and 37.5 at.% (*x* = 0.75) the crystallite size ranges of *d*_*c*_ ≥ *d*_*critical*_ = 7.8 nm and *d*_*c*_ ≥ *d*_*critical*_ = 9.1 nm were obtained, respectively. Details of the computational results delineating the contributions of surface and volume energy to the total energy can be found in the Supplementary Table S1.

The *ab initio* metastable phase formation predictions were critically appraised by comparison to experimental phase formation data obtained from combinatorially grown Ti_1−x_Al_x_N coatings with *x* = 0.05 to 0.86. Based on X-ray diffraction (XRD) data for *x* < 0.55 single phase c-TiAlN is formed, while for *x* > 0.69 single phase w-TiAlN is formed. For the intermediate concentration range of 0.55 ≤ *x* ≤ 0.69 the formation of a phase mixture was observed (relevant diffractograms can be found in the Supplementary Fig. S1). The composition-dependent crystallite size data (estimated with the Scherrer equation^23^) are depicted in Fig. 2(b) together with the values of *d*_*critical*_ predicted by DFT. The measured crystallite size is within 28 ± 2 nm for single phase c-Ti_1−x_Al_x_N (0.05 ≤ *x* ≤ 0.51). Upon formation of metastable w-Ti_1−x_Al_x_N, the cubic solid solution phase crystallite sizes decrease from 23 nm (*x* = 0.55) to 6 nm (*x* = 0.69). The w-Ti_1−x_Al_x_N crystallite sizes are significantly smaller than the values of c-Ti_1−x_Al_x_N and increase from 2 to 4 nm in an Al concentration range of *x* = 0.55 to 0.86. It is evident from Fig. 2(b) that all w-Ti_1−x_Al_x_N crystallite sizes are smaller than the predicted *d*_*critical*_ and - except for *x* = 0.69 - the c-Ti_1−x_Al_x_N crystallite size values are larger than *d*_*critical*_. Considering that crystallite size determination based on Scherrer’s equation provides a lower bound value since broadening contributions from the presence of structural defects, such as dislocations, grain boundaries and microstrains as well as coherency strains, are not taken into account, the observed agreement between theory and experiment is very good.

**Figure 3:**
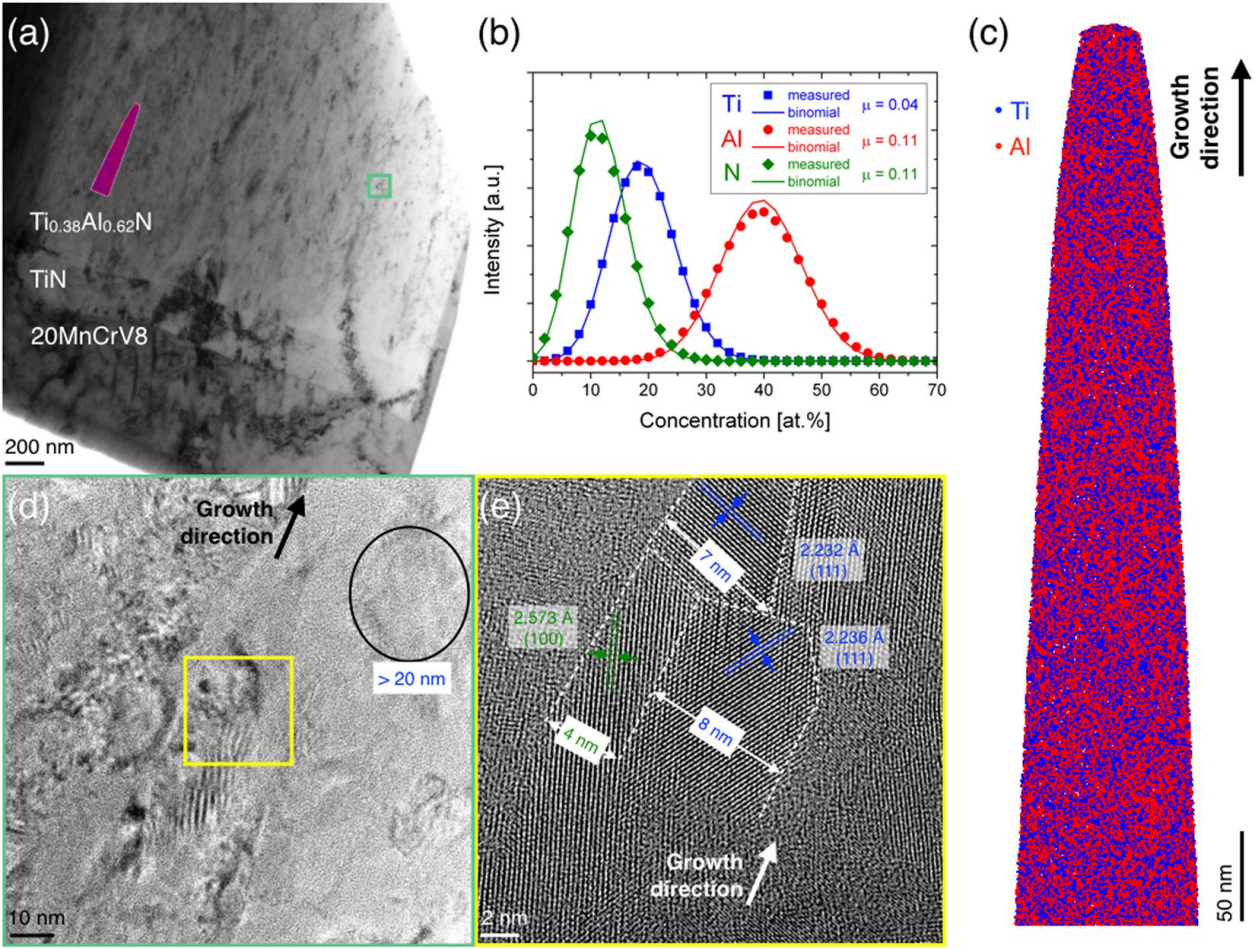
Spatially-resolved characterization of Ti_0.38_Al_0.62_N. (**a**) Transmission electron micrograph (TEM) showing the steel substrate, TiN interlayer and the Ti_0.38_Al_0.62_N coating. (**b**) Compositional distribution analysis of Ti (squares), Al (circles) and N (diamonds) and comparison to binomial, random distributions (lines with corresponding color code) within the (**c**) 3D atom probe tomography reconstruction. *μ* is the homogenization parameter with *μ* = 0 for a completely random distribution. (**d**) Higher magnification TEM covering the area of the box in (**a**) and (**e**) high resolution TEM covering the area of the box in (**d**).

The transmission electron micrograph (TEM) in Fig. 3(a) shows an overview of Ti_0.38_Al_0.62_N (1.8 μm thickness) deposited onto a 200 nm TiN interlayer on a 90MnCrV8 steel substrate. Consistent with the theoretical and experimental phase formation data, selected area electron diffraction data confirms the presence of c- and w-TiAlN solid solution phases and can be found in the Supplementary Fig. S2. Local chemical composition analysis was carried out in the region indicated by the atom probe tomography (APT) tip outline marked in Fig. 3(a) and the corresponding APT reconstruction is presented in Fig. 3(c). From the comparison of measured and calculated binomial (random) distributions of the constitutional elements in Fig. 3(b) it can be inferred that Ti, Al and N are distributed in a close to random fashion as indicated by the Pearson correlation coefficient *μ* which is close to zero. Thereby, the computational approach of c- and w-TiAlN comparison is validated.

The size and shape of several individual crystallites are depicted in Fig. 3(d,e). While a c-TiAlN crystallite with a size of >20 nm and sphere-like shape can be observed in Fig. 3(d), an elongated w-TiAlN crystallite with (100) orientation is adjacent to two elongated c-TiAlN crystallites with (111) orientation in Fig. 3(e). The crystallite width of 4 nm can be estimated for w-TiAlN, while the cubic crystallites exhibit widths of 7 and 8 nm and these values are close to the calculated critical crystallite size ≥7.8 nm, see Fig. 2. Hence, it is obvious that c-TiAlN domains are significantly larger than w-TiAlN and the size relationship between c- and w-TiAlN is in qualitative agreement with the XRD crystallite size data. Based on high resolution TEM data it is reasonable to assume that c- and w-TiAlN crystallites are distributed homogeneously throughout the Ti_0.38_Al_0.62_N coating with almost identical chemical composition since the APT reconstruction with volume of approximately 50 × 50 × 500 nm should contain different c- and w-TiAlN crystallites (the image in Fig. 3(e) covers a region of 24 × 24 nm).

## Conclusions

We have demonstrated that surface energy contributions to the total energy must be considered for metastable phase stability predictions and the extent of c- and w-TiAlN phase stability regions is predominated by the crystallite size, a parameter which has been overlooked in the past. The here reported results provide an explanation for the sobering disagreement between DFT predictions and experimentally observed critical Al solubilities in metastable cubic TiAlN coatings of the last ten years. Furthermore, it is evident that critical solubility predictions for the design of metastable materials are flawed, if the previously overlooked surface energy contribution to the total energy is not considered.

## Methods

### *Ab initio* calculations of critical crystallite sizes

Critical crystallite sizes *d*_*critical*_ for metastable cubic Ti_1−x_Al_x_N phase formation were calculated by density functional theory^24^. Within the Vienna *ab inito* simulation package, projector augmented wave potentials and the general gradient approximation were employed^25^. Full structural relaxations were performed with convergence obtained at 10^−3^ eV, while an energy cut-off of 500 eV was used. Brillouin zone integration was done with a Monkhorst-Pack k-point mesh of 6 × 6 × 3^26^ and total energies were treated with Blöchl-corrections^27^. 2 × 2 × 4 supercells with 128 atoms were employed for c-Ti_1−x_Al_x_N and the configuration with the minimum total energy was utilized (configuration C#3 in the original paper)^14^. Atomic coordinates of the employed relaxed supercells are provided in Supplementary Tables S2 to S7.

The total energy was minimized as a function of volume with the Birch-Murnaghan equation of states^28^ and this energy is referred to as volume energy *E*_*vol*_ in the following. Besides the volume energy, surface energies were explicitly considered: supercell slabs were created in the (001) and (0001) lattice plane for c- and w-Ti_1−x_Al_x_N, respectively, vacuum layers with a thickness of approximately 15 Å were introduced and the total energy *E*_*slab*_ was calculated. Both surfaces of the cubic slab were populated by metal and non-metal atoms and one surface of the wurtzite slab was populated by metal atoms, the opposite surface slab was terminated by non-metal atoms. While describing only the (001) and (0001) surface orientations is an approximation and, hence, a significant simplification of reality, the obtained predictions are consistent with the experimentally obtained critical solubility data indicating that the here selected orientations are relevant. The surface energy *E*_*surf*_ was obtained from the energy difference with respect to the surface area *A*_*surf*_ of the two surfaces created:

2 $${E}_{surf}=\frac{{E}_{slab}-{E}_{vol}}{2{A}_{surf}}$$

Therefore, the total energy *E*_*total*_ depends explicitly on *A*_*surf*_ which represents an energetic penalty for *E*_*total*_:

3 $${E}_{total}={E}_{vol}+{A}_{surf}{E}_{surf}$$

*E*_*vol*_ is normalized per atom [eV atom^−1^] and *E*_*surf*_ can be transformed from [J m^−2^] to [eV g atom^−1^ m^−2^] by taking the elementary charge *e* and Avogadro’s number *N*_*A*_ into account. Then, the product of specific *A*_*surf*_ [m^2^ g^−1^] and *E*_*surf*_ [eV g atom^−1^ m^−2^] results in [eV atom^−1^] and allows for addition of *E*_*vol*_ and *A*_*surf*_ *E*_*surf*_ in equation (3). Comparing c- and w-Ti_1−x_Al_x_N with *x* = 0.50, 0.625 and 0.75, a crossover of the total energy was obtained for each composition as presented in Fig. 2(a).

The critical crystallite size *d*_*critical*_ was calculated from the specific crossover surface area *A*_*surf-crossover*_, based on the assumption of cubic-shaped crystallites with an edge length *d* and the specific surface area *A*_*surf*_ = 6*d* ^2^/*m*. Combining the specific surface area with the density *ρ* = *m*/*V* and the volume *V* = *d* ^3^ results in equation (1). The variation of crystallite surface area determines the number of crystallites within the fixed volume. Density values were obtained from the equilibrium volume and Ti, Al and N atomic masses of 47.867, 26.9815 and 14.0067, respectively, weighted with the number of the respective atoms within the supercell. Critical crystallite sizes were calculated for Al concentrations of *x* = 0.50, 0.625 and 0.75 by determining *A*_*surf-crossover*_ at the crossover of *E*_*total,cubic*_ and *E*_*total,wurtzite*_.

### Coating synthesis

Ti_1−x_Al_x_N coatings were grown by cathodic arc evaporation in an industrial scale Oerlikon Balzers Ingenia p3e deposition system with six arc sources. Combinatorial synthesis^29^ was realized by employing three targets at different heights in the deposition chamber. The TiAl target compositions of (100/0), (50/50), (33/67) and (10/90) were combined differently in several growth experiments, resulting in an Al concentration range of *x* = 0.05 to 0.86 on the metal sublattice. 90MnCrV8 steel substrates with 23 mm diameter were mounted along the height of the two-fold substrate rotation carousel. The base pressure was always <3 × 10^−4^ Pa, the substrates were heated to 450 °C and surface contaminations were removed by plasma etching. Prior to deposition of Ti_1−x_Al_x_N, a TiN interlayer with approximately 200 nm thickness was applied by using three Ti targets. Subsequently, the Ti_1−x_Al_x_N layer was synthesized with a N_2_ deposition pressure of 3.5 Pa, a substrate bias potential of −40 V and coating thicknesses were in the range of 3 to 4 μm.

### Chemical composition analysis

Al/(Ti + Al) ratios were determined by energy dispersive X-ray spectroscopy (EDX) in a JEOL JSM-6480 scanning electron microscope with an EDAX Genesis 2000 detection system at 10 kV acceleration voltage.

Three dimensional compositional distributions on the nanometer scale were studied by atom probe tomography (APT) in a CAMECA LEAP 4000X HR. Laser-assisted field evaporation of Ti_0.38_Al_0.62_N was carried out with a laser energy of 30 pJ and a pulse frequency of 250 kHz. The tip temperature was kept at 60 K. APT specimens were prepared by focused ion beam (FIB) using a FEI HELIOS Nanolab 660 dual-beam microscope employing a standard lift-out procedure^30^.

### Crystal structure and crystallite size analysis

Coating crystal structures were measured by X-ray diffraction using a Siemens D5000 diffractometer in Bragg-Brentano geometry with Cu Kα radiation and the voltage and current were 40 kV and 40 mA, respectively. 2*θ* ranges of 20 to 80° were scanned at a step size of 0.01° and the measurement time was 2 s per step. Phase formation was studied by comparing lattice plane peaks with the *International Center for Diffraction Data* database using powder diffraction files (PDF) of face-centered cubic TiN (38–1420), AlN (25–1495), wurtzite AlN (25–1133) and body-centered cubic Fe (06–0696).

Crystallite sizes *D* were estimated from the (200) lattice plane peak in the diffractograms, based on the Scherrer equation^23^

4 $$D=2\sqrt{\frac{ln(2)}{\pi }}\frac{\lambda }{FWHM}\frac{1}{\cos \,\theta }$$

with *λ*, *FWHM* and *θ* being the X-ray wavelength, full width at half maximum and incidence angle, respectively.

The crystallite size distribution of Ti_0.38_Al_0.62_N was investigated by transmission electron microscopy (TEM). The TEM lamella was fabricated by FIB with a lift-out, mounted on an Omniprobe 3-posts copper grid and thinned to a thickness of <70 nm. Post-thinning was carried out by illumination with a 500 eV ion beam using a Fischione Nanomill device. Before imaging, the lamella and sample holder were cleaned in an O_2_ plasma with 12 eV energy for 1 minute for surface carbon contamination removal.

High resolution TEM was performed with an image-corrected FEI Titan 80–300 microscope operating in 300 kV with an information limit <100 pm^31^. The microscope was equipped with a field emission gun and capable to correct astigmatism, coma, star aberration and spherical aberration to the 3^rd^ order. Images were recorded with a 2 k × 2 k slow scan charged coupled Gatan UltraScan 1000 P camera system and the device controlling software of DigitalMicrograph.

### Data availability

The authors declare that all relevant data supporting the findings of this study are available within the paper and its Supplementary Information.

## Electronic supplementary material

Supplementary Information
