# Supplementary material for: Crystallite size-dependent metastable phase formation of TiAlN coatings
Source: Sci Rep. 2017 Nov 23;7:16096. doi: 10.1038/s41598-017-16567-z (PMC5700947; doi:10.1038/s41598-017-16567-z)
Supplement: Supplementary file 1 — Supplementary Information [file 41598_2017_16567_MOESM1_ESM.pdf]

Crystallite size-dependent metastable phase formation of TiAlN coatings

Marcus Hans*[1], Denis Music[1], Yen-Ting Chen[1]†, Lena Patterer[1], Anders O. Eriksson[2], Denis Kurapov[2], Jürgen Ramm[2], Mirjam Arndt[2], Helmut Rudigier[3] and Jochen M. Schneider[1]

[1]Materials Chemistry, RWTH Aachen University, Kopernikusstr. 10, D-52074 Aachen, Germany.

[2]Oerlikon Surface Solutions AG, Oerlikon Balzers, Iramali 18, LI-9496 Balzers, Liechtenstein.

[3]Oerlikon Surface Solutions AG, Oerlikon Balzers, Churer Str. 120, CH-8808 Pfäffikon, Switzerland.

*Correspondence and requests for materials should be addressed to M.H. (email: hans@mch.rwth-aachen.de)

†Present address: Center for Solvation Science, Ruhr-Universität Bochum, Universitätsstr. 150, D-44801 Bochum, Germany.

Contents of supplementary information:

1 Effect of volume and surface energy contributions on $d_{critical}$ ... 2

2 Crystal structure and phase fraction of w-TiAlN in $Ti_{1-x}Al_xN$ ... 3

3 Spatially-resolved crystal structure analysis ... 4

4 References ... 4

5 Atomic coordinates of c- and w-$Ti_{1-x}Al_xN$ supercells ... 5

1 Effect of volume and surface energy contributions on $d_{critical}$

Values of E_{vol}, E_{surf}, $d_{critical}$ and ρ are provided for c- and w-$Ti_{1-x}Al_xN$ (x = 0.50, 0.625 and 0.75) in table S1. The shift of the cross-over towards larger $d_{critical}$ values with increasing Al concentration can be affected by contributions of (i) E_{vol} and (ii) E_{surf}.

(i) The volume energy exhibits a strictly decreasing trend for increasing Al concentration. In case of c-$Ti_{1-x}Al_xN$, calculated E_{vol} values were -8.4, -8.1, -7.8 eV atom^{-1} for $x = 0.50$, 0.625, 0.75, respectively. Similarly, E_{vol} values of -8.2, -8.0, -7.7 eV atom^{-1} were calculated for w-$Ti_{1-x}Al_xN$ with $x = 0.50$, 0.625, 0.75, respectively. However, the average decrease of E_{vol} appears to be smaller for w-$Ti_{1-x}Al_xN$ ($\Delta E_{vol,w}$ = 248 meV atom^{-1}) compared to c-$Ti_{1-x}Al_xN$ ($\Delta E_{vol,c}$ = 310 meV atom^{-1}), resulting in smaller volume energy differences between c- and w-$Ti_{1-x}Al_xN$ for increasing x.

(ii) Surface energies of w-$Ti_{1-x}Al_xN$ are with 2.3, 1.6, 1.6 J m^{-2} for x = 0.50, 0.625, 0.75 always smaller than in case of c-$Ti_{1-x}Al_xN$ with 2.8, 3.0, 2.4 J m^{-2}. These E_{surf} values may be rationalized by a simple nearest-neighbor broken-bond model[1] and the nearest-neighbor broken-bond density of w-$Ti_{1-x}Al_xN$ is approximately 50% lower than the broken-bond density of corresponding c-$Ti_{1-x}Al_xN$. Moreover, the significant decrease of the w-$Ti_{1-x}Al_xN$ surface energy for Al concentrations $x > 0.50$ affects the shift of the cross-over towards smaller critical crystallite sizes.

x in $Ti_{1-x}Al_xN$	$E_{vol,c}$ [eV atom^{-1}]	$E_{surf,c}$ [J m^{-2}]	ρ_c [g cm^{-3}]	$E_{vol,w}$ [eV atom^{-1}]	$E_{surf,w}$ [J m^{-2}]	ρ_w [g cm^{-3}]	$d_{critical}$ [nm]
0.50	-8.413	2.797	4.694	-8.019	2.305	3.750	1.9
0.625	-8.013	2.975	4.519	-7.837	1.554	3.427	7.8
0.75	-7.793	2.353	4.345	-7.589	1.577	3.465	9.1

Table S1. Calculated values for E_{vol}, E_{surf}, ρ and $d_{critical}$ for c- and w- $Ti_{1-x}Al_xN$.

2 Crystal structure and phase fraction of w-TiAlN in $Ti_{1-x}Al_xN$

Phase formation data of c- and w-TiAlN in combinatorially grown $Ti_{1-x}Al_xN$ coatings are presented in Fig. S1 for different Al concentrations *x*. The c-TiAlN (200) lattice plane peak can be observed for Al concentrations < 0.73 in Fig. S1(a), while only the c-TiN interlayer (left-hand shoulder of the c-TiAlN peak for $x < 0.73$) is visible for higher Al concentrations. First indications for w-TiAlN formation are observed at $x = 0.55$ due to the broad peak in the 2θ range of 32 to 34°. Therefore, single phase c- and w-TiAlN is obtained for $x < 0.55$ and $x > 0.69$ and in between these concentrations a phase mixture of c- and w-TiAlN is present. The w-TiAlN phase fraction was calculated for all $Ti_{1-x}Al_xN$ coatings from the relative area below w-TiAlN lattice plane peaks with respect to c- and w-TiAlN and is shown in Fig. S1(b), emphasizing the single phase and phase mixed concentration ranges.

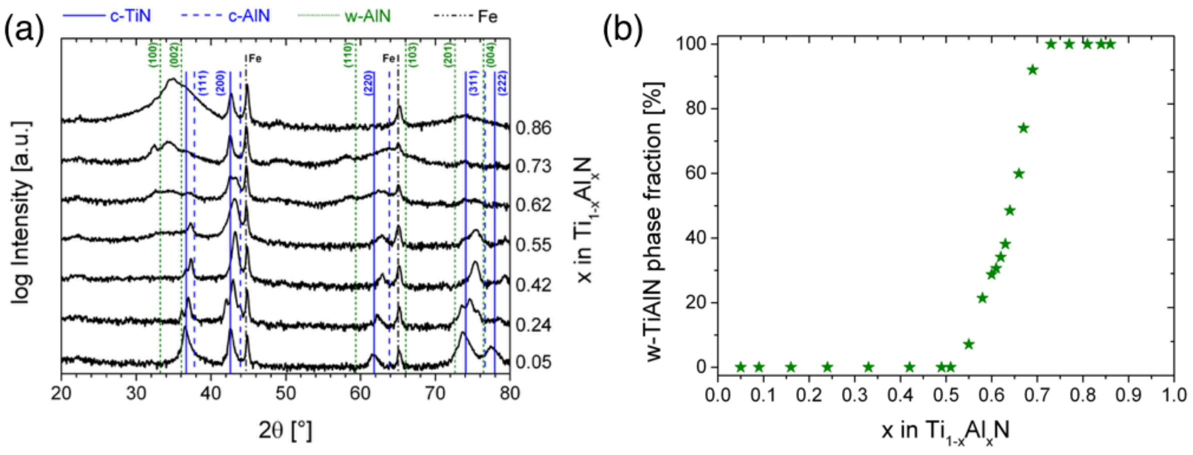

Figure S1. Experimental phase formation data of combinatorially grown $Ti_{1-x}Al_xN$. (**a**) Selected diffractograms of $Ti_{1-x}Al_xN$ for different Al concentrations *x*. Solid and dashed lines indicate reference lines for face-centered cubic TiN (PDF 38-1420), AlN (25-1495), short-dashed lines correspond to wurtzite AlN (PDF 25-1133) and dashed-dotted lines to body-centered cubic Fe (PDF card 06-0696). (**b**) w-TiAlN phase fraction calculated from the area below c- and w-TiAlN lattice plane peaks.

3 Spatially-resolved crystal structure analysis

The distribution of c- and w-TiAlN in $Ti_{0.38}Al_{0.62}N$ was investigated locally in the region indicated by the circle in Fig. S2(a). A phase mixture of c- and w-TiAlN can be identified from the ring-like diffraction segments within the selected area electron diffraction pattern in Fig. S2(b). Hence, spatially-resolved crystal structure analysis is in accordance with the integrated crystal structure analysis.

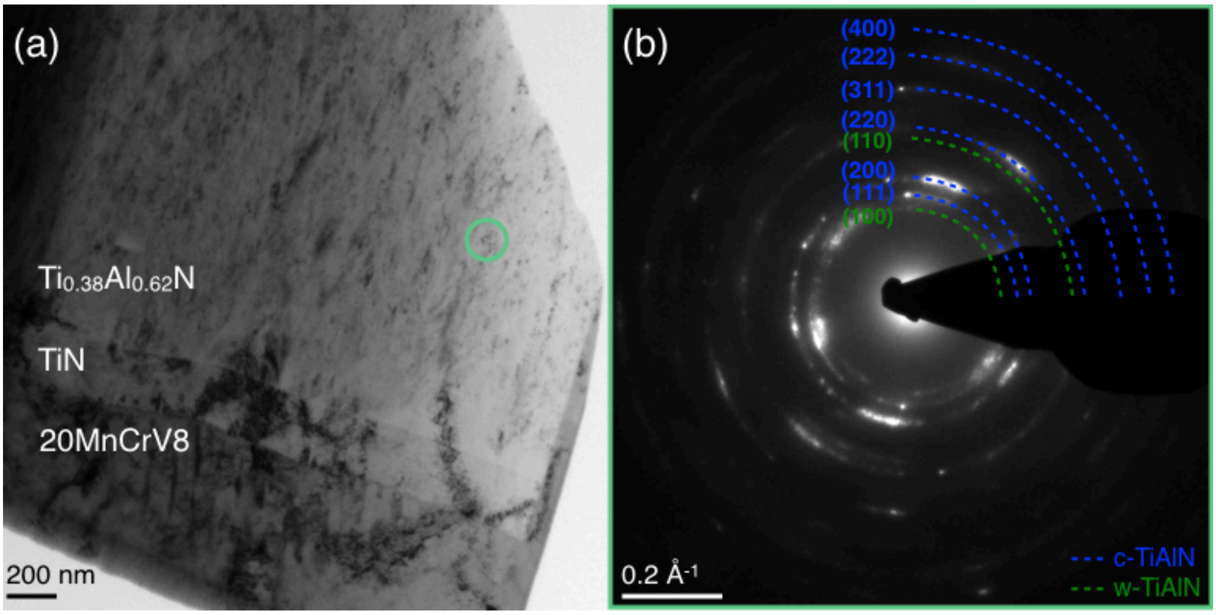

Figure S2. Spatially-resolved crystal structure analysis of $Ti_{0.38}Al_{0.62}N$. (**a**) Transmission electron micrograph (TEM) showing the steel substrate, TiN interlayer and the $Ti_{0.38}Al_{0.62}N$ coating. (**b**) Selected area electron diffraction pattern obtained from the region indicated by the circle in (**a**). c- and w-TiAlN lattice plane ring segments are indicated by blue and green dashed lines, respectively.

4 References

1. Holec, D. & Mayrhofer, P. H. Surface energies of AlN allotropes from first principles. *Scripta. Mater.* **67**, 760-762 (2012).

5 Atomic coordinates of c- and w-$Ti_{1-x}Al_xN$ supercells

In the following, fractional atomic coordinates of relaxed supercells for DFT calculations are presented in tables S2 to S7 and corresponding lattice parameters as well as supercell lattice vectors are provided in each table caption. c-$Ti_{1-x}Al_xN$ was described with a 2×2×4 supercell (128 atoms), while for w-$Ti_{1-x}Al_xN$ a 3×3×3 supercell (108 atoms) was used.

$c\text{-}Ti_{0.50}Al_{0.50}N$									
Metal sublattice					Non-metal sublattice				
Atom #	Type	x	y	z	Atom#	Type	x	y	z
1	Ti	0.00104	0.00091	0.00045	1	N	0.25718	0.25707	0.12851
2	Ti	0.50099	0.50089	0.00044	2	N	0.74481	0.24471	0.12238
3	Ti	0.00095	0.25091	0.12547	3	N	0.24480	0.74466	0.12236
4	Ti	0.50095	0.75093	0.12548	4	N	0.75720	0.75709	0.12851
5	Ti	0.25107	0.00081	0.12548	5	N	0.24480	0.50708	0.00351
6	Ti	0.75097	0.50088	0.12545	6	N	0.75718	0.49474	0.49737
7	Ti	0.25104	0.25091	0.00042	7	N	0.25722	0.99472	0.49738
8	Ti	0.75102	0.75092	0.00042	8	N	0.50720	0.24472	0.00352
9	Ti	0.50100	0.00089	0.25045	9	N	0.99484	0.25709	0.49738
10	Ti	0.00096	0.50087	0.25044	10	N	0.49484	0.75710	0.49738
11	Ti	0.50100	0.25087	0.37542	11	N	0.00719	0.74472	0.00350
12	Ti	0.00103	0.75084	0.37541	12	N	0.49478	0.49469	0.12851
13	Ti	0.75098	0.00088	0.37543	13	N	0.00716	0.50706	0.12237
14	Ti	0.25101	0.50086	0.37542	14	N	0.50722	0.00708	0.12238
15	Ti	0.75093	0.25083	0.25045	15	N	0.99482	0.99470	0.12851
16	Ti	0.25103	0.75089	0.25043	16	N	0.24481	0.24469	0.37236
17	Ti	0.00104	0.00091	0.50045	17	N	0.75720	0.25708	0.37852
18	Ti	0.50099	0.50089	0.50044	18	N	0.25721	0.75707	0.37852
19	Ti	0.00095	0.25091	0.62547	19	N	0.25715	0.49469	0.24737
20	Ti	0.50095	0.75093	0.62548	20	N	0.74479	0.50707	0.25351
21	Ti	0.25107	0.00081	0.62548	21	N	0.24479	0.00708	0.25352
22	Ti	0.75097	0.50088	0.62545	22	N	0.75720	0.99469	0.24738
23	Ti	0.25104	0.25091	0.50042	23	N	0.49478	0.25708	0.24737
24	Ti	0.75102	0.75092	0.50042	24	N	0.00716	0.24469	0.25351
25	Ti	0.50100	0.00089	0.75045	25	N	0.50719	0.74468	0.25351
26	Ti	0.00096	0.50087	0.75044	26	N	0.99482	0.75706	0.24737
27	Ti	0.50100	0.25087	0.87542	27	N	0.50718	0.50708	0.37237
28	Ti	0.00103	0.75084	0.87541	28	N	0.99483	0.49471	0.37851
29	Ti	0.75098	0.00088	0.87543	29	N	0.49484	0.99471	0.37854
30	Ti	0.25101	0.50086	0.87542	30	N	0.00720	0.00705	0.37238
31	Ti	0.75093	0.25083	0.75045	31	N	0.74485	0.74469	0.37237
32	Ti	0.25103	0.75089	0.75043	32	N	0.74484	0.00710	0.00353
33	Al	0.50103	0.00093	0.00046	33	N	0.25718	0.25707	0.62851
34	Al	0.00100	0.50090	0.00045	34	N	0.74481	0.24471	0.62238
35	Al	0.50102	0.25087	0.12545	35	N	0.24480	0.74466	0.62236
36	Al	0.00102	0.75086	0.12545	36	N	0.75720	0.75709	0.62851
37	Al	0.75100	0.00090	0.12547	37	N	0.24480	0.50708	0.50351
38	Al	0.25098	0.50088	0.12545	38	N	0.75718	0.49474	0.99737
39	Al	0.75101	0.25092	0.00044	39	N	0.25722	0.99472	0.99738
40	Al	0.25102	0.75090	0.00044	40	N	0.50720	0.24472	0.50352
41	Al	0.00100	0.00089	0.25045	41	N	0.99484	0.25709	0.99738
42	Al	0.50098	0.50088	0.25044	42	N	0.49484	0.75710	0.99738
43	Al	0.00100	0.25088	0.37543	43	N	0.00719	0.74472	0.50350
44	Al	0.50101	0.75090	0.37544	44	N	0.49478	0.49469	0.62851
45	Al	0.25103	0.00088	0.37544	45	N	0.00716	0.50706	0.62237
46	Al	0.75100	0.50088	0.37544	46	N	0.50722	0.00708	0.62238
47	Al	0.25099	0.25089	0.25044	47	N	0.99482	0.99470	0.62851
48	Al	0.75099	0.75090	0.25044	48	N	0.24481	0.24469	0.87236
49	Al	0.50103	0.00093	0.50046	49	N	0.75720	0.25708	0.87852
50	Al	0.00100	0.50090	0.50045	50	N	0.25721	0.75707	0.87852
51	Al	0.50102	0.25087	0.62545	51	N	0.25715	0.49469	0.74737
52	Al	0.00102	0.75086	0.62545	52	N	0.74479	0.50707	0.75351
53	Al	0.75100	0.00090	0.62547	53	N	0.24479	0.00708	0.75352
54	Al	0.25098	0.50088	0.62545	54	N	0.75720	0.99469	0.74738
55	Al	0.75101	0.25092	0.50044	55	N	0.49478	0.25708	0.74737
56	Al	0.25102	0.75090	0.50044	56	N	0.00716	0.24469	0.75351
57	Al	0.00100	0.00089	0.75045	57	N	0.50719	0.74468	0.75351
58	Al	0.50098	0.50088	0.75044	58	N	0.99482	0.75706	0.74737
59	Al	0.00100	0.25088	0.87543	59	N	0.50718	0.50708	0.87237
60	Al	0.50101	0.75090	0.87544	60	N	0.99483	0.49471	0.87851
61	Al	0.25103	0.00088	0.87544	61	N	0.49484	0.99471	0.87854
62	Al	0.75100	0.50088	0.87544	62	N	0.00720	0.00705	0.87238
63	Al	0.25099	0.25089	0.75044	63	N	0.74485	0.74469	0.87237
64	Al	0.75099	0.75090	0.75044	64	N	0.74484	0.00710	0.50353

Table S2. Atomic coordinates of $c\text{-}Ti_{0.50}Al_{0.50}N$ (a = 4.175 Å, $\vec{a} = \vec{b}$ = 8.350 Å, $\vec{c}$ = 16.700 Å).

$c\text{-}Ti_{0.375}Al_{0.625}N$									
Metal sublattice					Non-metal sublattice				
Atom #	Type	x	y	z	Atom#	Type	x	y	z
1	Ti	0.25092	0.99560	0.12277	1	N	0.24709	0.25376	0.12686
2	Ti	0.75114	0.50608	0.12817	2	N	0.75493	0.24798	0.12401
3	Ti	0.25105	0.24564	0.99775	3	N	0.24105	0.74162	0.12076
4	Ti	0.75096	0.75609	0.00314	4	N	0.76095	0.76018	0.13012
5	Ti	0.75109	0.00617	0.37809	5	N	0.25492	0.50378	0.00187
6	Ti	0.25087	0.49565	0.37272	6	N	0.74708	0.49800	0.49902
7	Ti	0.75101	0.25628	0.25309	7	N	0.26100	0.99160	0.49577
8	Ti	0.25100	0.74552	0.24779	8	N	0.50670	0.25208	0.00400
9	Ti	0.25092	0.99560	0.62277	9	N	0.99531	0.25796	0.50103
10	Ti	0.75114	0.50608	0.62817	10	N	0.49531	0.74969	0.49689
11	Ti	0.25105	0.24564	0.49775	11	N	0.00669	0.74381	0.99985
12	Ti	0.75096	0.75609	0.50314	12	N	0.49535	0.49381	0.12486
13	Ti	0.75109	0.00617	0.87809	13	N	0.00673	0.49971	0.12187
14	Ti	0.25087	0.49565	0.87272	14	N	0.50667	0.00796	0.12603
15	Ti	0.75101	0.25628	0.75309	15	N	0.99530	0.00206	0.12900
16	Ti	0.25100	0.74552	0.74779	16	N	0.24104	0.24160	0.37077
17	Ti	0.00106	0.00090	0.00046	17	N	0.76094	0.26015	0.38012
18	Ti	0.50100	0.75086	0.12546	18	N	0.24709	0.75380	0.37688
19	Ti	0.00099	0.50087	0.25044	19	N	0.26101	0.49154	0.24574
20	Ti	0.50097	0.25094	0.37543	20	N	0.74100	0.51025	0.25515
21	Ti	0.00106	0.00090	0.50046	21	N	0.25492	0.00378	0.25187
22	Ti	0.50100	0.75086	0.62546	22	N	0.74708	0.99800	0.24902
23	Ti	0.00099	0.50087	0.75044	23	N	0.49533	0.24969	0.24689
24	Ti	0.50097	0.25094	0.87543	24	N	0.00671	0.24378	0.24985
25	Al	0.50101	0.00089	0.00045	25	N	0.50667	0.75209	0.25400
26	Al	0.00101	0.50088	0.00044	26	N	0.99530	0.75799	0.25103
27	Al	0.50100	0.25090	0.12545	27	N	0.50664	0.50796	0.37603
28	Al	0.00101	0.75087	0.12544	28	N	0.99527	0.50206	0.37902
29	Al	0.75100	0.00516	0.12760	29	N	0.49535	0.99382	0.37486
30	Al	0.25100	0.49663	0.12331	30	N	0.00671	0.99971	0.37188
31	Al	0.75099	0.25518	0.00260	31	N	0.75492	0.74802	0.37403
32	Al	0.25100	0.74661	0.99829	32	N	0.74105	0.01016	0.00511
33	Al	0.00100	0.00089	0.25044	33	N	0.24709	0.25376	0.62686
34	Al	0.50101	0.50087	0.25045	34	N	0.75493	0.24798	0.62401
35	Al	0.00100	0.25090	0.37543	35	N	0.24105	0.74162	0.62076
36	Al	0.50100	0.75086	0.37544	36	N	0.76095	0.76018	0.63012
37	Al	0.25100	0.99663	0.37330	37	N	0.25492	0.50378	0.50187
38	Al	0.75099	0.50515	0.37759	38	N	0.74708	0.49800	0.99902
39	Al	0.25100	0.24664	0.24829	39	N	0.26100	0.99160	0.99577
40	Al	0.75099	0.75515	0.25259	40	N	0.50670	0.25208	0.50400
41	Al	0.50101	0.00089	0.50045	41	N	0.99531	0.25796	0.00103
42	Al	0.00101	0.50088	0.50044	42	N	0.49531	0.74969	0.99689
43	Al	0.50100	0.25090	0.62545	43	N	0.00669	0.74381	0.49985
44	Al	0.00101	0.75087	0.62544	44	N	0.49535	0.49381	0.62486
45	Al	0.75100	0.00516	0.62760	45	N	0.00673	0.49971	0.62187
46	Al	0.25100	0.49663	0.62331	46	N	0.50667	0.00796	0.62603
47	Al	0.75099	0.25518	0.50260	47	N	0.99530	0.00206	0.62900
48	Al	0.25100	0.74661	0.49829	48	N	0.24104	0.24160	0.87077
49	Al	0.00100	0.00089	0.75044	49	N	0.76094	0.26015	0.88012
50	Al	0.50101	0.50087	0.75045	50	N	0.24709	0.75380	0.87688
51	Al	0.00100	0.25090	0.87543	51	N	0.26101	0.49154	0.74574
52	Al	0.50100	0.75086	0.87544	52	N	0.74100	0.51025	0.75515
53	Al	0.25100	0.99663	0.87330	53	N	0.25492	0.00378	0.75187
54	Al	0.75099	0.50515	0.87759	54	N	0.74708	0.99800	0.74902
55	Al	0.25100	0.24664	0.74829	55	N	0.49533	0.24969	0.74689
56	Al	0.75099	0.75515	0.75259	56	N	0.00671	0.24378	0.74985
57	Al	0.50100	0.50089	0.00046	57	N	0.50667	0.75209	0.75400
58	Al	0.00100	0.25089	0.12545	58	N	0.99530	0.75799	0.75103
59	Al	0.50101	0.00091	0.25046	59	N	0.50664	0.50796	0.87603
60	Al	0.00099	0.75087	0.37544	60	N	0.99527	0.50206	0.87902
61	Al	0.50100	0.50089	0.50046	61	N	0.49535	0.99382	0.87486
62	Al	0.00100	0.25089	0.62545	62	N	0.00671	0.99971	0.87188
63	Al	0.50101	0.00091	0.75046	63	N	0.75492	0.74802	0.87403
64	Al	0.00099	0.75087	0.87544	64	N	0.74105	0.01016	0.50511

Table S3. Atomic coordinates of $c\text{-}Ti_{0.375}Al_{0.625}N$ (a = 4.156 Å, $\vec{a} = \vec{b}$ = 8.311 Å, $\vec{c}$ = 16.622 Å).

c-$Ti_{0.25}Al_{0.75}N$									
Metal sublattice					Non-metal sublattice				
Atom #	Type	x	y	z	Atom#	Type	x	y	z
1	Ti	0.25101	0.00092	0.12543	1	N	0.25107	0.25775	0.12886
2	Ti	0.75098	0.50091	0.12543	2	N	0.75092	0.24403	0.12203
3	Ti	0.25101	0.25096	0.00040	3	N	0.25093	0.74402	0.12203
4	Ti	0.75102	0.75088	0.00045	4	N	0.75108	0.75776	0.12887
5	Ti	0.75102	0.00086	0.37547	5	N	0.25092	0.50778	0.00387
6	Ti	0.25100	0.50083	0.37546	6	N	0.75108	0.49402	0.49701
7	Ti	0.75098	0.25091	0.25043	7	N	0.25109	0.99402	0.49703
8	Ti	0.25100	0.75082	0.25047	8	N	0.50602	0.25093	0.00043
9	Ti	0.25101	0.00092	0.62543	9	N	0.99599	0.25087	0.50046
10	Ti	0.75098	0.50091	0.62543	10	N	0.49600	0.75087	0.50046
11	Ti	0.25101	0.25096	0.50040	11	N	0.00603	0.75092	0.00043
12	Ti	0.75102	0.75088	0.50045	12	N	0.49598	0.50092	0.12542
13	Ti	0.75102	0.00086	0.87547	13	N	0.00599	0.50085	0.12546
14	Ti	0.25100	0.50083	0.87546	14	N	0.50602	0.00086	0.12547
15	Ti	0.75098	0.25091	0.75043	15	N	0.99599	0.00092	0.12543
16	Ti	0.25100	0.75082	0.75047	16	N	0.25093	0.24399	0.37201
17	Al	0.50100	0.00092	0.00046	17	N	0.75108	0.25774	0.37886
18	Al	0.00102	0.50089	0.00044	18	N	0.25110	0.75776	0.37887
19	Al	0.50099	0.25089	0.12544	19	N	0.25106	0.49401	0.24701
20	Al	0.00101	0.75087	0.12544	20	N	0.75091	0.50774	0.25386
21	Al	0.75101	0.00089	0.12545	21	N	0.25092	0.00773	0.25386
22	Al	0.25098	0.50087	0.12543	22	N	0.75108	0.99403	0.24704
23	Al	0.75101	0.25091	0.00045	23	N	0.49598	0.25086	0.25046
24	Al	0.25101	0.75088	0.00044	24	N	0.00600	0.25091	0.25043
25	Al	0.00101	0.00089	0.25045	25	N	0.50601	0.75091	0.25043
26	Al	0.50099	0.50089	0.25045	26	N	0.99598	0.75085	0.25046
27	Al	0.00101	0.25089	0.37544	27	N	0.50601	0.50085	0.37546
28	Al	0.50099	0.75088	0.37544	28	N	0.99599	0.50092	0.37542
29	Al	0.25101	0.00089	0.37545	29	N	0.49600	0.00092	0.37543
30	Al	0.75099	0.50088	0.37544	30	N	0.00603	0.00084	0.37547
31	Al	0.25098	0.25089	0.25044	31	N	0.75093	0.74401	0.37201
32	Al	0.75099	0.75088	0.25044	32	N	0.75094	0.00778	0.00387
33	Al	0.50100	0.00092	0.50046	33	N	0.25107	0.25775	0.62886
34	Al	0.00102	0.50089	0.50044	34	N	0.75092	0.24403	0.62203
35	Al	0.50099	0.25089	0.62544	35	N	0.25093	0.74402	0.62203
36	Al	0.00101	0.75087	0.62544	36	N	0.75108	0.75776	0.62887
37	Al	0.75101	0.00089	0.62545	37	N	0.25092	0.50778	0.50387
38	Al	0.25098	0.50087	0.62543	38	N	0.75108	0.49402	0.99701
39	Al	0.75101	0.25091	0.50045	39	N	0.25109	0.99402	0.99703
40	Al	0.25101	0.75088	0.50044	40	N	0.50602	0.25093	0.50043
41	Al	0.00101	0.00089	0.75045	41	N	0.99599	0.25087	0.00046
42	Al	0.50099	0.50089	0.75045	42	N	0.49600	0.75087	0.00046
43	Al	0.00101	0.25089	0.87544	43	N	0.00603	0.75092	0.50043
44	Al	0.50099	0.75088	0.87544	44	N	0.49598	0.50092	0.62542
45	Al	0.25101	0.00089	0.87545	45	N	0.00599	0.50085	0.62546
46	Al	0.75099	0.50088	0.87544	46	N	0.50602	0.00086	0.62547
47	Al	0.25098	0.25089	0.75044	47	N	0.99599	0.00092	0.62543
48	Al	0.75099	0.75088	0.75044	48	N	0.25093	0.24399	0.87201
49	Al	0.00105	0.00092	0.00046	49	N	0.75108	0.25774	0.87886
50	Al	0.50099	0.50090	0.00045	50	N	0.25110	0.75776	0.87887
51	Al	0.00100	0.25089	0.12544	51	N	0.25106	0.49401	0.74701
52	Al	0.50100	0.75088	0.12544	52	N	0.75091	0.50774	0.75386
53	Al	0.50100	0.00089	0.25046	53	N	0.25092	0.00773	0.75386
54	Al	0.00098	0.50088	0.25045	54	N	0.75108	0.99403	0.74704
55	Al	0.50100	0.25089	0.37545	55	N	0.49598	0.25086	0.75046
56	Al	0.00104	0.75086	0.37544	56	N	0.00600	0.25091	0.75043
57	Al	0.00105	0.00092	0.50046	57	N	0.50601	0.75091	0.75043
58	Al	0.50099	0.50090	0.50045	58	N	0.99598	0.75085	0.75046
59	Al	0.00100	0.25089	0.62544	59	N	0.50601	0.50085	0.87546
60	Al	0.50100	0.75088	0.62544	60	N	0.99599	0.50092	0.87542
61	Al	0.50100	0.00089	0.75046	61	N	0.49600	0.00092	0.87543
62	Al	0.00098	0.50088	0.75045	62	N	0.00603	0.00084	0.87547
63	Al	0.50100	0.25089	0.87545	63	N	0.75093	0.74401	0.87201
64	Al	0.00104	0.75086	0.87544	64	N	0.75094	0.00778	0.50387

Table S4. Atomic coordinates of c-$Ti_{0.25}Al_{0.75}N$ (a = 4.134 Å, $\vec{a} = \vec{b}$ = 8.268 Å, $\vec{c}$ = 16.536 Å).

w-$Ti_{0.50}Al_{0.50}N$									
Metal sublattice					Non-metal sublattice				
Atom #	Type	x	y	z	Atom#	Type	x	y	z
1	Ti	0.95401	0.01145	0.00400	1	N	0.95235	0.02111	0.13357
2	Ti	0.62446	0.95617	0.01975	2	N	0.25599	0.98509	0.12359
3	Ti	0.49339	0.31103	0.00170	3	N	0.65316	0.99757	0.15156
4	Ti	0.05299	0.67540	0.00204	4	N	0.15721	0.33497	0.12468
5	Ti	0.72161	0.66224	0.95785	5	N	0.48850	0.32558	0.13701
6	Ti	0.45198	0.08185	0.14162	6	N	0.83601	0.34409	0.12000
7	Ti	0.99668	0.46098	0.17051	7	N	0.04414	0.68584	0.13500
8	Ti	0.66275	0.47999	0.13003	8	N	0.36008	0.65070	0.11905
9	Ti	0.54056	0.76231	0.15785	9	N	0.72781	0.71255	0.09314
10	Ti	0.95116	0.00409	0.33249	10	N	0.12919	0.12467	0.29385
11	Ti	0.63191	0.97974	0.27906	11	N	0.44650	0.09235	0.28678
12	Ti	0.50637	0.31785	0.27031	12	N	0.79341	0.14415	0.29387
13	Ti	0.03587	0.69306	0.33386	13	N	0.00014	0.44968	0.29886
14	Ti	0.70812	0.66148	0.32863	14	N	0.32666	0.44215	0.28736
15	Ti	0.46565	0.08753	0.50499	15	N	0.68166	0.45233	0.27643
16	Ti	0.99639	0.45826	0.49450	16	N	0.22550	0.78268	0.29465
17	Ti	0.65856	0.45483	0.51043	17	N	0.53316	0.75834	0.29161
18	Ti	0.55129	0.75308	0.51518	18	N	0.85171	0.80904	0.28235
19	Ti	0.96020	0.00310	0.66668	19	N	0.95208	0.01348	0.46343
20	Ti	0.64447	0.96644	0.65362	20	N	0.27740	0.98997	0.45755
21	Ti	0.49159	0.31206	0.66069	21	N	0.64399	0.98198	0.49931
22	Ti	0.04223	0.68085	0.65487	22	N	0.16498	0.33353	0.45948
23	Ti	0.72673	0.64800	0.68291	23	N	0.49581	0.31436	0.48934
24	Ti	0.46314	0.09169	0.84237	24	N	0.82302	0.33729	0.45880
25	Ti	0.96626	0.47671	0.82510	25	N	0.05118	0.68887	0.47090
26	Ti	0.64826	0.43650	0.83068	26	N	0.37455	0.64330	0.45989
27	Ti	0.56253	0.75285	0.81158	27	N	0.73244	0.66849	0.45565
28	Al	0.26830	0.98743	0.00083	28	N	0.13377	0.12271	0.62518
29	Al	0.15413	0.33658	0.99806	29	N	0.45016	0.08569	0.63312
30	Al	0.83042	0.33494	0.99689	30	N	0.80614	0.13155	0.62601
31	Al	0.37730	0.65459	0.99392	31	N	0.99496	0.44204	0.62559
32	Al	0.12312	0.11988	0.17027	32	N	0.32007	0.43959	0.62979
33	Al	0.80082	0.14401	0.17012	33	N	0.66978	0.43239	0.63802
34	Al	0.32165	0.44545	0.16176	34	N	0.23534	0.78590	0.62506
35	Al	0.21936	0.78138	0.16953	35	N	0.54227	0.74891	0.64712
36	Al	0.87708	0.80815	0.15767	36	N	0.85921	0.80692	0.62690
37	Al	0.28157	0.99460	0.33189	37	N	0.95147	0.01444	0.79643
38	Al	0.16450	0.33636	0.33388	38	N	0.27485	0.99498	0.79466
39	Al	0.82861	0.34792	0.33493	39	N	0.62987	0.99724	0.79117
40	Al	0.37268	0.64887	0.33496	40	N	0.14632	0.33852	0.79423
41	Al	0.12834	0.11542	0.50035	41	N	0.47083	0.30817	0.79170
42	Al	0.79514	0.12737	0.50344	42	N	0.81947	0.31704	0.79355
43	Al	0.33293	0.43531	0.50460	43	N	0.06708	0.69094	0.78239
44	Al	0.23052	0.78183	0.50110	44	N	0.36881	0.64034	0.79502
45	Al	0.88114	0.80196	0.49946	45	N	0.76901	0.65176	0.81724
46	Al	0.28003	0.99183	0.66969	46	N	0.12520	0.12477	0.95784
47	Al	0.15491	0.33425	0.66983	47	N	0.44474	0.08124	0.97533
48	Al	0.83393	0.33179	0.67125	48	N	0.78696	0.12102	0.96370
49	Al	0.36949	0.64708	0.67272	49	N	0.99194	0.45249	0.95919
50	Al	0.12155	0.12356	0.83348	50	N	0.32371	0.43731	0.95994
51	Al	0.78418	0.11226	0.83777	51	N	0.66096	0.43720	0.97094
52	Al	0.31882	0.43334	0.83350	52	N	0.22519	0.78094	0.95546
53	Al	0.23139	0.78194	0.83125	53	N	0.53810	0.76346	0.94909
54	Al	0.91608	0.80084	0.83063	54	N	0.90803	0.80583	0.95256

Table S5. Atomic coordinates of w-$Ti_{0.50}Al_{0.50}N$ (a = 3.199 Å, c = 5.140 Å, $\vec{a}$ = 9.597 Å, $\vec{b}$ = 8.311 Å, $\vec{c}$ = 15.421 Å).

w-$Ti_{0.375}Al_{0.625}N$									
Metal sublattice					Non-metal sublattice				
Atom #	Type	x	y	z	Atom#	Type	x	y	z
1	Ti	0.95132	0.01928	0.99780	1	N	0.95000	0.00983	0.13522
2	Ti	0.77725	0.62931	0.97590	2	N	0.26692	0.99629	0.12955
3	Ti	0.44715	0.10350	0.15784	3	N	0.62435	0.97766	0.13812
4	Ti	0.97853	0.46472	0.13047	4	N	0.15451	0.33302	0.12634
5	Ti	0.94977	0.00669	0.32663	5	N	0.46753	0.32135	0.12457
6	Ti	0.61445	0.98662	0.32596	6	N	0.80356	0.30982	0.12770
7	Ti	0.72738	0.65742	0.32672	7	N	0.07753	0.67815	0.13136
8	Ti	0.64843	0.45114	0.47209	8	N	0.40515	0.65827	0.13092
9	Ti	0.55256	0.77182	0.46948	9	N	0.74859	0.66059	0.14095
10	Ti	0.95803	0.99699	0.66888	10	N	0.12262	0.12648	0.29581
11	Ti	0.72112	0.65375	0.60437	11	N	0.43554	0.11192	0.29466
12	Ti	0.45455	0.09731	0.83153	12	N	0.78829	0.12466	0.29603
13	Ti	0.95664	0.47395	0.83809	13	N	0.98161	0.44644	0.28837
14	Ti	0.47292	0.31933	0.97531	14	N	0.31320	0.44794	0.29209
15	Ti	0.08328	0.65231	0.97876	15	N	0.64441	0.43241	0.30841
16	Ti	0.64446	0.44917	0.12503	16	N	0.23488	0.79113	0.29231
17	Ti	0.06084	0.65857	0.31687	17	N	0.54031	0.76360	0.30559
18	Ti	0.45339	0.09553	0.49200	18	N	0.90677	0.79100	0.28964
19	Ti	0.06136	0.68858	0.67565	19	N	0.95437	0.00827	0.46260
20	Ti	0.65001	0.44981	0.83730	20	N	0.26564	0.98875	0.46179
21	Al	0.27687	0.99824	0.00089	21	N	0.63616	0.01297	0.46041
22	Al	0.14808	0.33731	0.99707	22	N	0.14956	0.33512	0.46301
23	Al	0.80292	0.30962	0.99841	23	N	0.46691	0.31802	0.45840
24	Al	0.40827	0.66686	0.99979	24	N	0.83338	0.33266	0.46002
25	Al	0.11848	0.12399	0.16687	25	N	0.06164	0.66358	0.45889
26	Al	0.78092	0.10596	0.16869	26	N	0.37207	0.64234	0.46179
27	Al	0.31947	0.44760	0.16203	27	N	0.75390	0.66878	0.46426
28	Al	0.24293	0.78299	0.16263	28	N	0.12350	0.12868	0.62944
29	Al	0.91090	0.78925	0.15541	29	N	0.44974	0.10954	0.63132
30	Al	0.27448	0.99887	0.33217	30	N	0.78001	0.10909	0.62957
31	Al	0.14646	0.33911	0.33189	31	N	0.97431	0.44909	0.63361
32	Al	0.81900	0.33698	0.32842	32	N	0.31897	0.44908	0.63377
33	Al	0.37354	0.65270	0.33229	33	N	0.64262	0.43044	0.62627
34	Al	0.12034	0.12163	0.49896	34	N	0.23761	0.79089	0.62890
35	Al	0.79386	0.12304	0.49978	35	N	0.55377	0.78275	0.61863
36	Al	0.31727	0.43721	0.50225	36	N	0.89536	0.78803	0.62005
37	Al	0.22765	0.77609	0.49815	37	N	0.94951	0.01454	0.80603
38	Al	0.91111	0.79285	0.49035	38	N	0.27316	0.00264	0.79350
39	Al	0.27805	0.00414	0.66320	39	N	0.62316	0.97990	0.79029
40	Al	0.14191	0.34513	0.67009	40	N	0.14580	0.34297	0.79919
41	Al	0.80703	0.32841	0.66507	41	N	0.46985	0.32503	0.80347
42	Al	0.39512	0.66473	0.66120	42	N	0.80177	0.31311	0.79513
43	Al	0.12074	0.12886	0.83198	43	N	0.08671	0.68094	0.81425
44	Al	0.77521	0.10568	0.83170	44	N	0.41278	0.66235	0.79365
45	Al	0.31375	0.45727	0.82882	45	N	0.77026	0.65792	0.82610
46	Al	0.25899	0.79097	0.83483	46	N	0.13062	0.12608	0.96097
47	Al	0.92426	0.79982	0.83962	47	N	0.45027	0.07956	0.96964
48	Al	0.61355	0.98863	0.65766	48	N	0.76548	0.10137	0.96256
49	Al	0.47864	0.32702	0.66686	49	N	0.96430	0.43611	0.97868
50	Al	0.59093	0.76999	0.83584	50	N	0.30313	0.46625	0.96117
51	Al	0.47342	0.32647	0.32763	51	N	0.64570	0.43867	0.97953
52	Al	0.99075	0.45403	0.50112	52	N	0.26139	0.79344	0.96216
53	Al	0.57544	0.76456	0.17347	53	N	0.58005	0.76490	0.96486
54	Al	0.61342	0.97167	0.00569	54	N	0.92353	0.80804	0.97292

Table S6. Atomic coordinates of w-$Ti_{0.375}Al_{0.625}N$ (a = 3.205 Å, c = 4.840 Å, $\vec{a}$ = 9.615 Å, $\vec{b}$ = 8.327 Å, $\vec{c}$ = 14.520 Å).

w-$Ti_{0.25}Al_{0.75}N$									
Metal sublattice					Non-metal sublattice				
Atom #	Type	x	y	z	Atom#	Type	x	y	z
1	Ti	0.94822	0.00525	0.98777	1	N	0.94621	0.99977	0.12647
2	Ti	0.72942	0.65340	0.98213	2	N	0.26997	0.98836	0.12682
3	Ti	0.44612	0.11155	0.16065	3	N	0.61678	0.98514	0.13155
4	Ti	0.99513	0.44971	0.14169	4	N	0.17473	0.32583	0.12630
5	Ti	0.93880	0.99646	0.33054	5	N	0.50079	0.33614	0.12891
6	Ti	0.62050	0.98936	0.33379	6	N	0.81321	0.31859	0.12708
7	Ti	0.72321	0.67905	0.34174	7	N	0.06498	0.67596	0.12503
8	Ti	0.65628	0.45440	0.47852	8	N	0.38979	0.66150	0.12871
9	Ti	0.55432	0.77069	0.48006	9	N	0.72573	0.65826	0.13467
10	Ti	0.94854	0.00659	0.66394	10	N	0.12072	0.12009	0.29462
11	Ti	0.71639	0.65923	0.61404	11	N	0.43946	0.10997	0.29176
12	Ti	0.44769	0.11252	0.82706	12	N	0.78786	0.12866	0.28954
13	Ti	0.99138	0.45109	0.83277	13	N	0.99857	0.45203	0.28871
14	Al	0.27455	0.99715	0.99878	14	N	0.32689	0.44427	0.29329
15	Al	0.16585	0.33568	0.99742	15	N	0.65648	0.43533	0.30299
16	Al	0.81318	0.32456	0.00101	16	N	0.22215	0.78042	0.29509
17	Al	0.38523	0.66603	0.99993	17	N	0.53498	0.77032	0.30197
18	Al	0.11828	0.11250	0.16628	18	N	0.90669	0.78255	0.28928
19	Al	0.78366	0.10819	0.16309	19	N	0.95461	0.00697	0.46786
20	Al	0.33818	0.44711	0.16637	20	N	0.28042	0.99267	0.46232
21	Al	0.23101	0.77816	0.16722	21	N	0.61798	0.01361	0.46511
22	Al	0.89800	0.78044	0.16173	22	N	0.15712	0.33237	0.46024
23	Al	0.27381	0.99407	0.33225	23	N	0.47383	0.31682	0.45931
24	Al	0.15597	0.33467	0.32962	24	N	0.83952	0.33167	0.45900
25	Al	0.83192	0.34017	0.32984	25	N	0.05974	0.66454	0.46056
26	Al	0.37309	0.65407	0.33453	26	N	0.36858	0.64686	0.46158
27	Al	0.12517	0.11964	0.49793	27	N	0.75445	0.67699	0.47549
28	Al	0.78813	0.12083	0.49746	28	N	0.12470	0.12719	0.62782
29	Al	0.32503	0.43902	0.49870	29	N	0.44479	0.11159	0.63124
30	Al	0.22653	0.77747	0.49962	30	N	0.77754	0.11494	0.62766
31	Al	0.91443	0.79353	0.49943	31	N	0.99436	0.45296	0.63127
32	Al	0.27516	0.99783	0.66407	32	N	0.32679	0.44821	0.62906
33	Al	0.15936	0.33943	0.66672	33	N	0.65380	0.42948	0.62076
34	Al	0.81787	0.33455	0.66385	34	N	0.22200	0.78369	0.62878
35	Al	0.38197	0.66400	0.66267	35	N	0.54359	0.78211	0.62450
36	Al	0.12130	0.11700	0.83187	36	N	0.90461	0.79135	0.62880
37	Al	0.77775	0.10886	0.83083	37	N	0.94560	0.00146	0.80088
38	Al	0.33983	0.44991	0.83143	38	N	0.27114	0.99135	0.79288
39	Al	0.23270	0.77957	0.83170	39	N	0.61600	0.98868	0.79095
40	Al	0.89788	0.78034	0.82970	40	N	0.16768	0.33330	0.79672
41	Al	0.60529	0.99205	0.66031	41	N	0.49901	0.33534	0.79137
42	Al	0.48754	0.32947	0.66085	42	N	0.81989	0.31883	0.79217
43	Al	0.05517	0.66841	0.66697	43	N	0.06330	0.67177	0.79565
44	Al	0.66835	0.44338	0.82884	44	N	0.39109	0.66280	0.79302
45	Al	0.56566	0.77247	0.82963	45	N	0.73096	0.65743	0.81201
46	Al	0.48298	0.32492	0.33253	46	N	0.12757	0.12565	0.95899
47	Al	0.05641	0.66221	0.33015	47	N	0.44535	0.11243	0.96736
48	Al	0.44849	0.10833	0.50056	48	N	0.76766	0.10942	0.96067
49	Al	0.00059	0.45120	0.49884	49	N	0.99327	0.44868	0.97505
50	Al	0.66876	0.44498	0.17290	50	N	0.33363	0.45134	0.96081
51	Al	0.56207	0.76862	0.17146	51	N	0.65559	0.43142	0.95795
52	Al	0.60325	0.99013	0.00113	52	N	0.22797	0.78343	0.96017
53	Al	0.48986	0.33039	0.99962	53	N	0.55113	0.78036	0.96085
54	Al	0.05975	0.67155	0.99779	54	N	0.90270	0.78292	0.96000

Table S7. Atomic coordinates of w-$Ti_{0.25}Al_{0.75}N$ (a = 3.213 Å, c = 4.933 Å, $\vec{a}$ = 9.640 Å, $\vec{b}$ = 8.348 Å, $\vec{c}$ = 14.798 Å).
